# Correction: Silica cubosomes templated by a star polymer

**DOI:** 10.1039/c9ra90064k

**Published:** 2019-09-02

**Authors:** Congcong Cui, Lu Han, Shunai Che

**Affiliations:** School of Chemical Science and Engineering, Tongji University 1239 Siping Road Shanghai 200092 China luhan@tongji.edu.cn; School of Chemistry and Chemical Engineering, State Key Laboratory of Metal Matrix Composites, Shanghai Jiao Tong University 800 Dongchuan Road Shanghai 200240 P. R. China

## Abstract

Correction for ‘Silica cubosomes templated by a star polymer’ by Congcong Cui *et al.*, *RSC Adv.*, 2019, **9**, 6118–6124.

The authors wish to report extra characterisation data to provide further evidence for the synthesis of the AB_2_ star polymer reported in the original article.

The authors have added additional ^1^H NMR (Fig. S8) and MALDI-TOF MS (Fig. S9) spectra for all synthesis steps of the reaction to support the polymer synthesis. The ^1^H NMR spectra show the appearance and disappearance of two of the characteristic peaks for 4-methylphenyl in the reactions to synthesise PEG–N–OH_2_ (marked in the green box, Fig. S8). Subsequently, the characteristic methyl peak of the macroinitiator (PEG–N–Br_2_) is also observed (marked in the blue box, Fig. S8). The MALDI-TOF MS spectrum of the product of each reaction shows a set of peaks with a spacing of 44 *m*/*z* (corresponding to the PEG repeating unit, –CH_2_–CH_2_–O–). The main peak position changes as the terminal group changes. The absolute molecular weight of each product obtained through MALDI-TOF MS matches well with the calculated molecular weight.

The electronic supplementary information (ESI) of the original article has been updated to reflect these changes.

In addition, the authors regret that the signal for the CH–Br group in the ^1^H NMR spectrum, shown in Fig. 1 of the original article, was incorrectly assigned. The signal should have been assigned to the broad peak at around 4.5 ppm. Due to the small number of end groups compared to the polymer chain, the signals of the end groups are very weak and difficult to identify. The correct version of Fig. 1 is shown below.

**Fig. 1:**
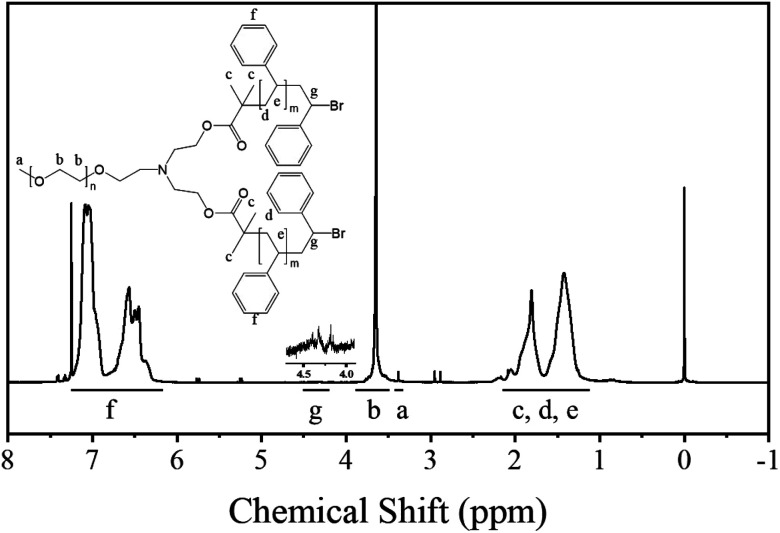
^1^H NMR spectrum of the polymer.

The Royal Society of Chemistry apologises for these errors and any consequent inconvenience to authors and readers.

## Supplementary Material

